# Curcumin-Loaded HA-Based Nerve Guidance Conduit with PLA Microfibers-Filled Lumen Enhances Schwann Cell Proliferation and Alignment

**DOI:** 10.1021/acsomega.5c10443

**Published:** 2026-02-19

**Authors:** Sandra Fuster-Gómez, Arantxa Martínez-Férriz, Francisco Navarro-Páez, Manuel Monleón Pradas

**Affiliations:** † Center for Biomaterials and Tissue Engineering, 16774 Universitat Politècnica De València, C. de Vera s/n, Valencia 46022, Spain; ‡ Networking Research Center on Bioengineering, Biomaterials and Nanomedicine, Valencia 46022, Spain; ■ Biomedical Research Networking Center on Bioengineering, Biomaterials and Nanomedicine (CIBER-BBN), Valencia 46022, Spain

## Abstract

Peripheral nerve injuries (PNIs) represent a significant health challenge for society, as they can result in lifelong disabilities. Despite the rapid advancement of tissue engineering technology, the regeneration of peripheral nerves through nerve guidance conduits (NGCs) remains less favorable than the gold standard treatment, nerve autografts. Consequently, improving the effectiveness of NGCs is essential. The objective of this study was to develop a more effective nerve guidance conduit. To achieve this, hyaluronic acid tubular scaffolds were loaded with curcumin, which has been shown to promote peripheral nerve regeneration, and filled with polylactic acid microfibers to encourage directed growth. A physicochemical characterization of this novel NGC was performed to ensure a thorough understanding of its structural features and performance characteristics. The impact of curcumin and PLA microfibers on Schwann cells, a crucial player in nerve regeneration, was evaluated. The sustained release of curcumin from the hyaluronic hydrogel was observed to enhance the proliferation of Schwann cells, which aligned themselves in a parallel fashion with the PLA microfibers. The proliferation and alignment of these cells within the NGC provides an optimal environment for the regeneration of nerves.

## Graphical abstract

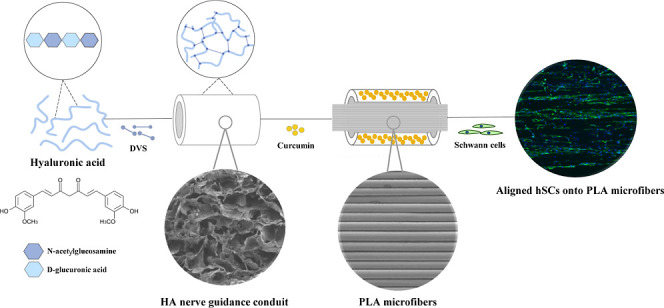

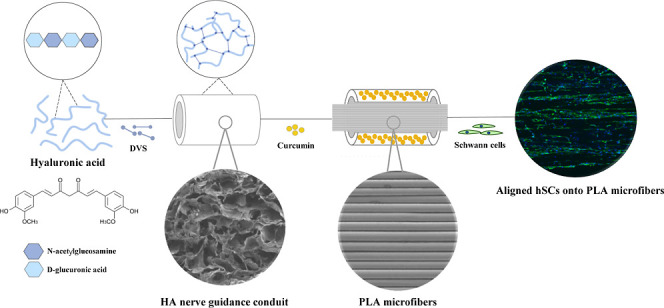

## 1 Introduction

Peripheral nerve injuries (PNIs) represent a significant health and socio-economic challenge for society, as they can result in lifelong disabilities.^1,2^ Autografts are currently considered the gold standard for critical nerve defects in the medical clinics.^3^ Nevertheless, autografts have some drawbacks such as, comorbidity caused by additional surgery, limited availability of donor nerve and the dimension mismatches.^1^ Consequently, research efforts have been made to address these limitations, resulting in the development of nerve guidance conduits (NGCs). These tube-shaped scaffolds serve as a bridge between the damaged nerve endings.^4^

While there are many types of NGCs available and some have been approved by regulatory agencies, their ability to promote nerve regeneration is not as effective as autografts, highlighting a need for improvement.^5^ These improvements should focus on a multidisciplinary approach, providing the NGCs with topological and biochemical cues to promote the oriented growth of nerve fibers and to modulate cellular behavior.^1,4,6^ The first step for the fabrication of NGCs involves the selection of an appropriate material, which can be either synthetic or natural. Among the available options, hyaluronic acid (HA), a naturally occurring glycosaminoglycan found in the extracellular matrix, stands out as a suitable option due to its positive attributes.^7^ HA therapeutic benefits on neuronal regeneration include minimal immune response, high biocompatibility, and good biodegradability, reduce scar formation.^7–11^ Furthermore, its mechanical properties closely resemble those of soft nerve tissues, making it an excellent material for NGCs.^11^

Schwann cells (SCs), the main glial cells of the peripheral nervous system (PNS), serve as support cells for neurons. As a result, they are essential for the survival and function of neurons.^12^ With this in mind, it is not surprising that they also have a crucial role in nerve regeneration.^13^ SCs during the first days after injury, proliferate and create Büngner bands (longitudinal cell strands)^14^ which allow the guided elongation of growth cones from proximal end to distal end.

Therefore, to enhance NGCs performance, it is crucial to imitate the native regenerating nerve tissue architecture more accurately, aiding in the creation of a cellular aligned pathway. Many researchers have turned to topological cues in order to accomplish this. An easy approach involves inserting microfibers directly into the NGC lumen.^6^ A wide variety of materials can be used to obtain microfibers but polylactic acid (PLA) has gained popularity over the past years, especially for neural regeneration.^15,16^ PLA is a synthetic polymer, frequently used in the field of tissue engineering due to its biocompatibility and biodegradability.^17^ The positive attributes of the material have led to its approval for use by both the FDA and the EMA.^18^

The release of bioactive factors, to modulate cellular behavior, is another method of enhancing the performance of NGCs, particularly in terms of cell proliferation and survival. Curcumin (Cur), a hydrophobic low-molecular-weight polyphenol compound extracted from *Curcuma longa*, has gained popularity over the years due to its various pharmacological effects, especially for regenerative medicine and tissue engineering.^19^ In addition, studies have demonstrated curcumin’s significant capacity for aiding in the repair of damaged PNS.^20–22^ However, there is a need for new curcumin formulations and/or improved delivery platforms, such as hydrogels, due to its low bioavailability and short biological half-life.^21^

To address these challenges, in the current study, we design a hyaluronic acid nerve guidance conduit loaded with curcumin and filled with polylactic acid microfibers. A comprehensive physicochemical evaluation of the NGC here developed was conducted. Moreover, human Schwann cells seeded inside the NGC aligned parallel to the PLA microfibers resembling bands of Büngner and the sustained release of curcumin from the hyaluronic hydrogel improved their proliferation, thus providing a favorable environment for nerve regeneration.

## 2 Materials and Methods

### 2.1 Hyaluronic Acid Nerve Guidance Conduit Preparation, Curcumin Loading and Polylactic Acid Microfiber Lain Insertion

The synthesis of hyaluronic acid-based nerve guidance conduits (HA-NGCs) was carried out as previously described.^11,23–25^ Briefly, HA (sodium salt from *Streptococcus equi* 1.5–1.8 MDa; 53746, Sigma-Aldrich) was prepared at a 5% concentration in 0.2 M sodium hydroxide (S8045, Sigma-Aldrich) and gently stirred for 24 h at room temperature. Afterward, the HA solution was cross-linked with divinyl sulfone (DVS-V3700, Sigma-Aldrich) in a 9:10 DVS/HA monomeric unit molar ratio, this mixture was magnetically stirred and pipetted into a mold. The mold is made of PTFE formed by tubular channels with an interior diameter of 3.50 mm, into which a PTFE fiber (3400175, Scharlab) with an outer diameter of 1.75 mm was introduced into the center of each channel to form the conduit’s lumen. At both ends of the PCL fiber, a PTFE stopper was inserted to maintain the PCL fiber’s position within the center. Then, the HA-DVS mixture inside the mold was frozen and then lyophilized (LyoQuest-85, Telstar Life Science) overnight at 0.01 mbar and −80 °C. After lyophilization, the HA-NGCs were hydrated in distilled water for 2 h, and the PCL fiber were extracted, HA-NGCs were cut to the desired length (5 mm in this case). Then, they were washed three times with deionized water for 30 min to ensure that any residue of unreacted DVS and NaOH was eliminated. Finally, size-cut HA conduits were lyophilized (Figure 1A–C) as described above and 1 mM of curcumin (C1386, Sigma-Aldrich) dissolved in ethanol was pipetted onto the HA-NGC xerogel (HA-Cur-NGCs) (Figure 1D–F).

**Figure 1:**
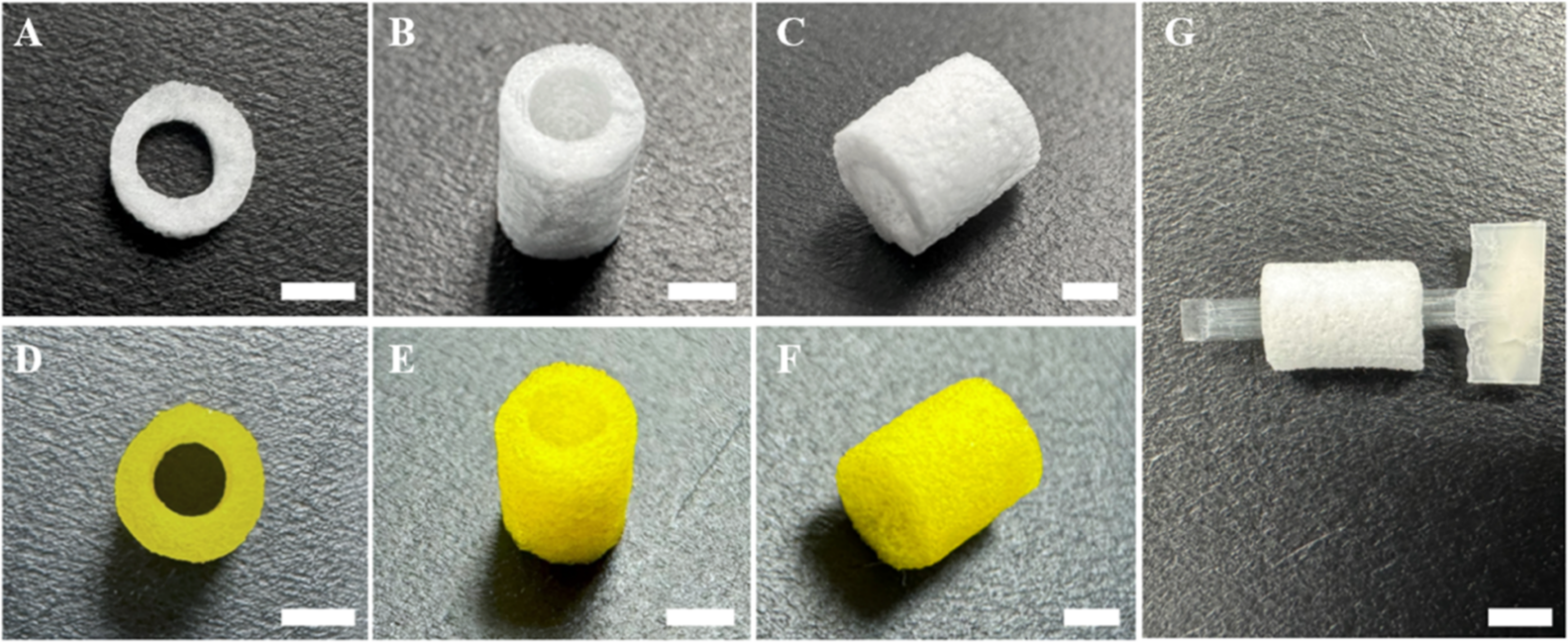
Representative macroscopic HA and curcumin-loaded HA nerve guidance conduits. (A) Top view, (B) transversal view and (C) longitudinal view of HA NGCs. (D) Top view, (E) transversal view and (F) longitudinal view of curcumin-loaded HA-NGCs (Cur-HA-NGCs). (G) HA-NGC with PLA microfiber bundle within the lumen. Scale bar 2 mm.

Then an aligned PLA fiber (Aitex Textile Research Institute) bundle comprising 66 microfibers with a diameter of 10 μm was positioned within the lumen of the HA-NGCs. To maintain the fiber bundle together, one end was thermally fused together, while the other was held together by a PCL band (Figure 1G). The PCL band allowed for better manipulation and ensured the bundle did not slip out of the HA-NGCs.

### 2.2 Morphological and Physicochemical Characterization

#### 2.2.1 High-Resolution Field Emission Scanning Electron Microscopy

Surface morphology of HA nerve guidance conduits with or without PLA microfibrils was examined by field emission scanning electron microscopy (HRFESEM - ZEISS, GeminiSEM 500). Samples were frozen with liquid nitrogen and axial and transversal cuts were made. Subsequently, the samples were placed onto a carbon tape and due to the thickness of the sample a carbon bridge was created between the sample and the carbon tape. Finally, the samples were sputter-coated with a thin layer of gold. Images were taken using a 4 kV acceleration voltage and 9.2 mm working distance.

#### 2.2.2 Thermogravimetric Analysis

Thermal degradation profiles of the HA- and HA-Cur-NGCs (*n* = 3) was conducted, a thermogravimetric analyzer (TGA/SDTA851, Mettler-Toledo operated by the STARexx software) was used. A heating ramp was applied from 30 to 800 °C at a heating rate of 10 °C/min with a nitrogen flux of 50 mL/min. As a result, thermograms where the samples’ residual weight is represented as a function of temperature were obtained and the most representative thermogram of each sample was plotted. The curves were normalized by dividing through the present weight at the 130 °C point of the heating ramp, point at which all the water absorbed by the HA- and HA-Cur-NGCs conduits was evaporated.

#### 2.2.3 Fourier Transform Infrared Spectroscopy Analysis

To investigate the chemical groups of the HA conduits loaded with or without curcumin (*n* = 3), FTIR was used (Cary 630 FTIR spectrometer, Agilent Technologies) in the attenuated total reflection mode. The spectra were obtained by averaging 24 scans at a resolution of 4 cm^–1^, spanning the range of 400–4000 cm^–1^. An air measurement was conducted under identical conditions and used as a baseline to correct background signal. The most representative spectrum was represented.

### 2.3 Curcumin Release Profile Characterization

The curcumin release from HA-Cur-NGCs was evaluated by incubating the samples (*n* = 6) in 1 mL of PBS (pH 7.4-21-031-CM, Corning). All samples were kept at 37 °C in a mini rocker-shaker (MR-1, Biosan) under constant agitation. After different incubation time periods the release medium was collected, replaced with the same volume of fresh medium and analyzed using a Nanodrop (ND-1000) at 426 nm in triplicate.

### 2.4 Cell Culture Assays

#### 2.4.1 Determination of Half-Maximal Inhibitory Concentration (IC_50_)

To establish the IC_50_ of curcumin and work at safe and noncytotoxic doses for hSCs cells, a colorimetric MTS assay (CellTiter 96 Aqueous One Solution Cell Proliferation Assay, Promega) was performed. To do so, hSCs (P10351, Innoprot) at passage 5 were seeded at a density of 1 × 10^4^ cells/well in a 96-well plate and allowed to attach for 24 h at 37 °C in a 5% CO_2_ atmosphere. After 24 h, cell medium (Schwann Cell Medium kit—P60123, Innoprot) was replaced with fresh medium supplemented with varying concentrations of curcumin (0–80 μM) (*n* = 4 per concentration). A positive control for cytotoxicity was carried out using dimethyl sulfoxide (DMSO, 472301, Honeywell) at a 0.5% (v/v) concentration. Following a 24 h incubation period, cell viability was determined following manufacturer’s instructions. Briefly, cells were rinsed with PBS, after which the reactant solution was added and incubated for 3 h at 37 °C and 5% CO_2_. The absorbance was determined with a spectrophotometer at a wavelength of 490 nm (Victor Multilabel Counter 1420, PerkinElmer).

#### 2.4.2 Cell Seeding within Conduits HA-Nerve Guidance Conduits

Prior to cell seeding within the HA-NGCs, samples were sterilized using γ radiation at 25 kGy (Aragogamma, Barcelona). Afterward, HA conduits were loaded with 10 μM of curcumin and lyophilized under sterile conditions. The PLA microfiber bundle was sterilized and sanitized by exposing the bundle to UV light on each side, and washed with 70° ethanol (ET0002025S, Scharlau) for 10 min and then washed with sterile Milli-Q water for 10 min each, to avoid any cytotoxic effect of the ethanol. Then, using tweezers, the microfiber bundle was inserted through the NGCs lumen. Finally, samples (HA- and HA-Cur-NGCs with PLA microfibers) were preconditioned by immersion in Dulbecco’s modified eagle’s medium (DMEM; high glucose (4.5 g/L); 10313021, ThermoFisher Scientific) supplemented with 1% penicillin/streptomycin (15140122, ThermoFisher Scientific) and 5% fetal bovine serum (FBS; 10270106, Gibco) and incubated overnight at 37 °C and 5% CO_2_. Before cell seeding, the preconditioning medium was removed from the samples, which were then air-dried inside the laminar flow cabinet to minimize overflow of the seeded cell suspension. At passage 5, hSCs were seeded at a density of 1 × 10^5^ within the HA- and HA-Cur-NGCs. This was achieved by inserting an extralong 10 μL micropipette tip (23-0011S, Biologix) at each end of the conduit to ensure homogeneous cell distribution in the lumen and were maintained in the incubator for 30 min before adding the SC medium to avoid cells from flowing out. The cells were kept at the same temperature and atmosphere parameters as mentioned above. The culture medium was renewed every 2 days.

#### 2.4.3 Proliferation Assay

To evaluate the effect of curcumin on hSCs proliferation inside the conduits, an MTS was performed (*n* = 5) at different cell culture intervals (1, 3, and 6 days). The assay was performed according to the manufacturer’s instructions. Briefly, at specified time points, the HA- and HA-Cur-NGCs were transferred on a new P48 cell culture plate and rinsed three times with PBS. Then, the reactant solution was added to each well. The samples were incubated for 2 h. Subsequently, five 100 μL aliquots from each sample were pipetted into a 96-well plate. Absorbance was measured as mentioned in Section 2.4.1.

#### 2.4.4 Immunostaining

hSCs morphology after culture within the HA- and HA-Cur-NGCs with PLA microfibers was observed by immunofluorescence staining. After the different cell culture time points (12 h and 1, 3, and 6 days), the samples were rinsed three times with cold PBS and fixed with 4% paraformaldehyde (PFA—47608, Sigma-Aldrich) for 15 min at room temperature. After fixation, the samples were washed three times with PBS for 5 min each to remove PFA residues. Then, cell membrane was permeabilized and nonspecific binding sites were blocked using a blocking buffer composed of 1 M PB (P3619, Sigma-Aldrich) diluted to 0.1 M with Milli-Q water and supplemented with 1% normal goat serum (NGS—50062Z, Thermo Fisher) and 0.1% Tween20 (P1379, Sigma-Aldrich) for 1 h at room temperature. Cell cytoskeleton actin filaments were stained with FITC-Phalloidin (B607, Life Technologies) diluted 1:400 in blocking buffer overnight at 4 °C. To acquire immunofluorescence images of the stained cells inside the lumen, the HA- and HA-Cur-NGCs were carefully cut open with a razor blade to reveal their interior. Finally, the samples were mounted using Fluoroshield mounting medium with DAPI (ab104139, Abcam). Samples were observed under a confocal microscope (Stellaris 8 falcon, Leica Microsystems).

#### 2.4.5 Nuclei Cell Counting

Cell nuclei were quantified from fluorescence micrographs using the StarDist plugin for Fiji/ImageJ.^26^ This tool automatically detects and segment individual nuclei, the total number of detected nuclei per image (*n* = 3 per condition) was calculated from the segmentation. All images were analyzed under identical acquisition and processing settings to ensure consistency.

### 2.5 Statistical Analysis

Results are expressed as mean ± standard deviation (SD). Statistical analysis was performed using GraphPad Prism 9.4 Software. Data normality was assessed using the Shapiro–Wilk test. The results were analyzed using one-way ANOVA. Multiple comparisons were performed using Tukey’s multiple-comparisons analysis if significant differences were determined. Significance was assigned at *p < 0.05; **p < 0.01; ***p < 0.001; ****p < 0.0001.

## 3 Results and Discussion

### 3.1 Hyaluronic Acid-Based Nerve Guidance Conduits Physicochemical Characterization

Morphological analysis of the HA-NGC was performed using HRSEM. The HRSEM images of a transversal cut (Figure 2A,B) of a HA-NGC and a longitudinal cut (Figure 2C) revealed a porous structure of the HA, which was consistent with the findings of previous published studies by the research group.^11,16,25^ Upon close examination, the pore size and density in the outer wall surface (Figure 2C’) differs from that of lumen’s inner wall (Figure 2C’’). The pore size and density are observed to be smaller in the lumen’s inner wall surface. This discrepancy in pore dimensions allows the diffusion of molecules into the HA-NGC, while simultaneously impeding cell migration out of the HA-NGC, as assessed in previously published results.^23,24^ The internal diameter of the HA-NGC xerogels’ was 2.1 ± 0.1 mm, while their external diameter was 3.9 ± 0.1 mm. The dimensions of the fully hydrated state of the HA-NGCs were 4.1 ± 0.2 mm in external diameter and 2.3 ± 0.1 mm in internal diameter (Table 1). The dimensions were selected for future in vivo studies using an injured sciatic nerve rat model.^27^ However, the length dimensions can be modified by cutting the hydrated HA tubular scaffold longitudinally to the desired size after demolding. Furthermore, the lumen diameter can be altered by using different-sized PCL fibers to create the internal cylindrical channel. The HRSEM images also showed that the PLA microfibers (Figure 2D) displayed a strongly aligned distribution, crucial for achieving a high degree of cell alignment as observed in previous studies.^15^

**Figure 2:**
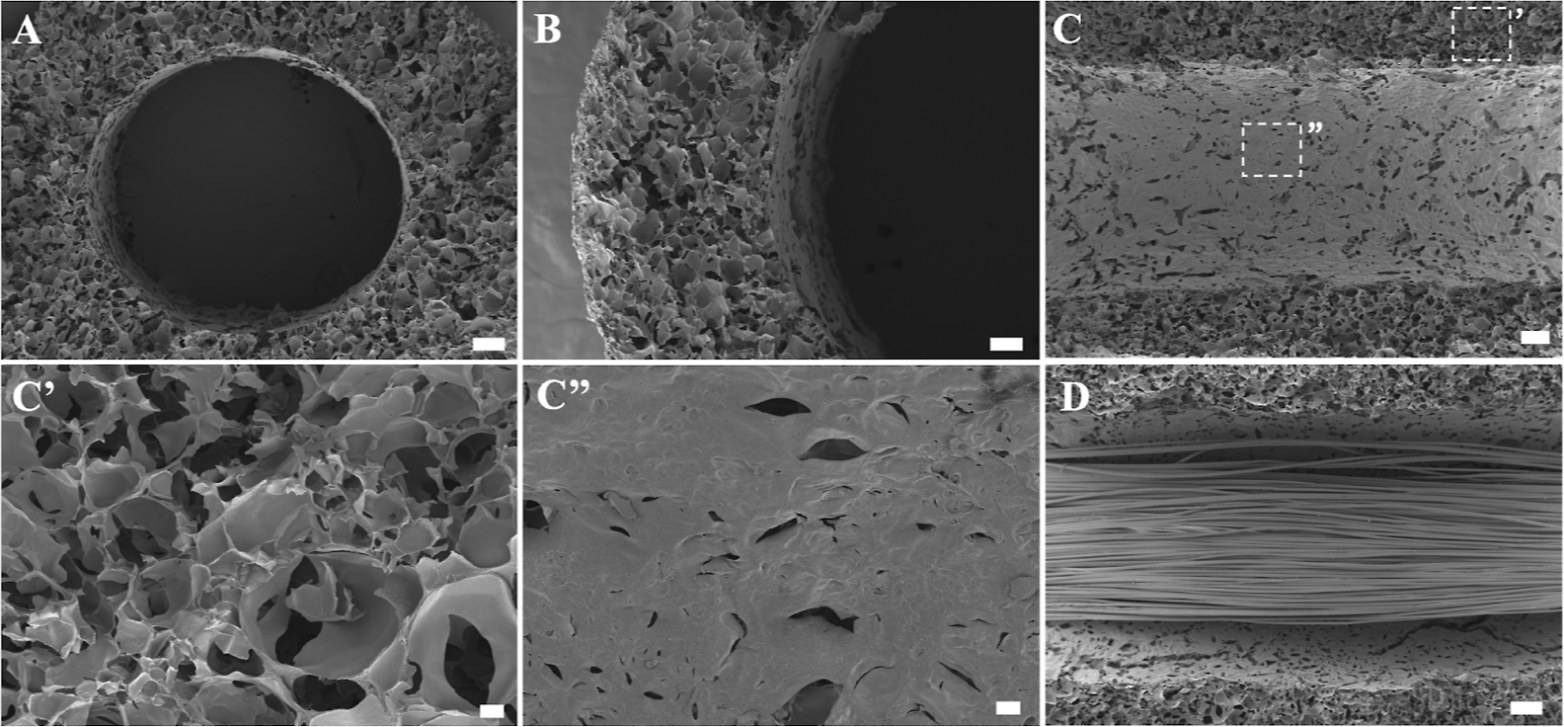
Structure and morphology of HA-NGCs and HA-NGCs with PLA microfibers. Scanning electron microscope images (HRSEM) from (A,B) an axial cut of HA-NGCs, (C) a longitudinal section, dividing the HA-NGCs into two parts to better visualize the surface of the lumen and a detail (C’,C’’) of the two different types of porosity in the tubes and (D) aligned PLA fiber bundle positioned within the lumen of the HA-NGC. Scale bars: (A) 200 μm, (B) 100 μm, (C) 200 μm, (C’,C’’) 20 μm, (D) 200 μm.

**Table 1:** Dimensions of the HA-Based Nerve Guidance Conduits (HA-NGC)^a^

sample type	internal diameter (mm)	external diameter (mm)
xerogel HA-NGC	2.1 ± 0.1	3.9 ± 0.1
fully hydrated HA-NGC	2.3 ± 0.1	4.1 ± 0.2

a Values are expressed as mean ± SD (*n* = 3).

The number of microfibers used per conduit was selected to balance topographical guidance and lumen permeability. A bundle of 66 PLA microfibers was found to span the inner diameter of the conduit while maintaining a largely open cross-sectional area, thus ensuring sufficient space for nutrient diffusion and Schwann cell proliferation, and alignment. Previous studies have shown that excessive intraluminal packing can hinder regeneration by physically restricting cellular infiltration and axonal extension.^28^ In this context, the selected bundle density was intentionally kept in the low packing-density regime in order to provide aligned topographical guidance without compromising free space for effective nerve regeneration. Valuable future work will address the systematic evaluation of bundle size and packing density to further elucidate their influence on Schwann cell behavior and subsequent axonal regeneration.

Thermal stability of HA-NGCs with or without Cur was evaluated by TGA (Figure 3). The degradation of HA involves two-step process that are characteristic of the breakdown of polysaccharides.^29^ There is an initial notable decrease in weight, reaching 45% at approximately 200 °C, followed by a smaller loss of 10% at temperatures around 500 °C. On the other hand, Cur is characterized by single-step degradation process with an initial weight loss around 210 °C. This degradation entails the dihydroxylation of the hydroxyl groups present in the curcumin molecules.^30,31^ Curcumin-loaded HA-NGCs (HA-Cur-NGCs) decompose in a similar fashion as HA conduits, however, there is a slightly higher final residual weight, due to the Cur content (26.8 ± 2.5% and 28.4 ± 1.8% at 700 °C, for HA-NGCs and HA-Cur-NGCs, respectively).

**Figure 3:**
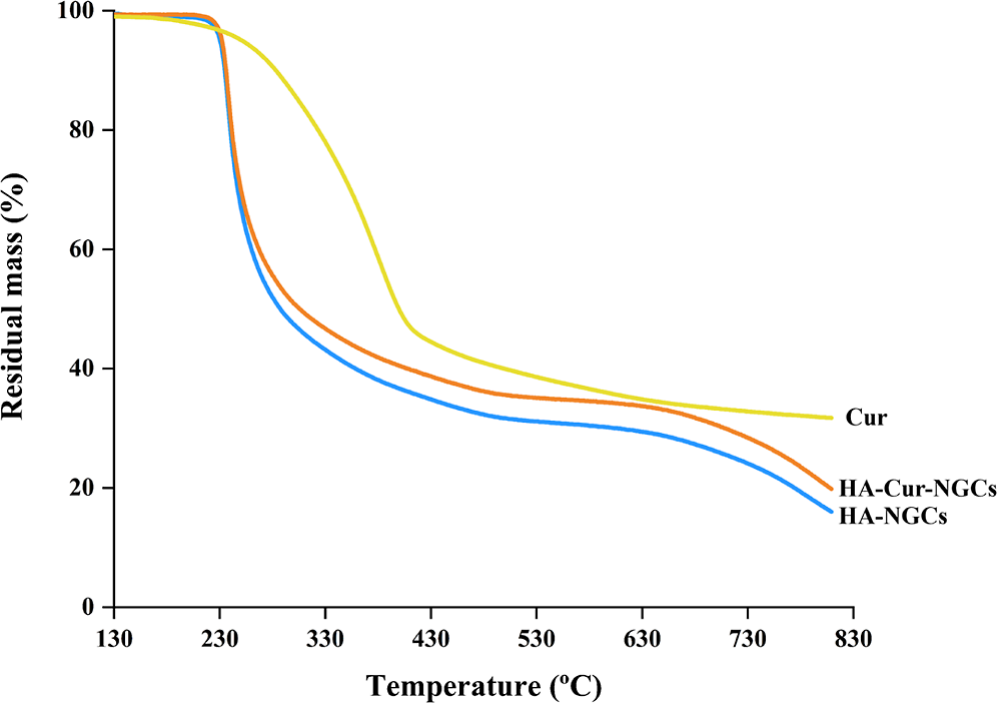
Nerve guidance conduits thermograms. Thermogravimetric curves (TGA) of Curcumin (Cur, yellow), hyaluronic acid-based nerve guidance conduits (HA-NGCs, blue) and curcumin-loaded hyaluronic acid nerve guidance conduits (HA-Cur-NGCs, orange) showing the residual mass as a function of temperature. Results shown are representative of *n* = 3 independent replicates.

FTIR analysis was conducted to chemically characterize HA-NGCs and HA-Cur-NGCs, confirming the existence of functional groups through the identification of distinct peaks in both samples (Figure 4A). Both samples presented HA characteristic peaks, with bands at 3300 cm^–1^ (O–H and N–H stretching vibrations); 2885 cm^–1^ (C–H stretching vibrations); 1606 cm^–1^ and 1404 cm^–1^ (asymmetric C=O and symmetric C–O stretching); and 1030 cm^–1^ (C–OH stretching vibrations).^32^ For the HA-Cur-NGCs (Figure 4B), an additional peak related with curcumin was encountered at 1510 cm^–1^ (C–C stretching of aromatic ring of curcumin).^33^ The FTIR analysis did not detect any new peaks, indicating that no new substantial interactions between HA or Cur were observed as a result of chemical or physical bonding. Consequently, it can be postulated that curcumin is retained within the HA matrix exclusively due to its porous nature, which is in accordance with its release profile.

**Figure 4:**
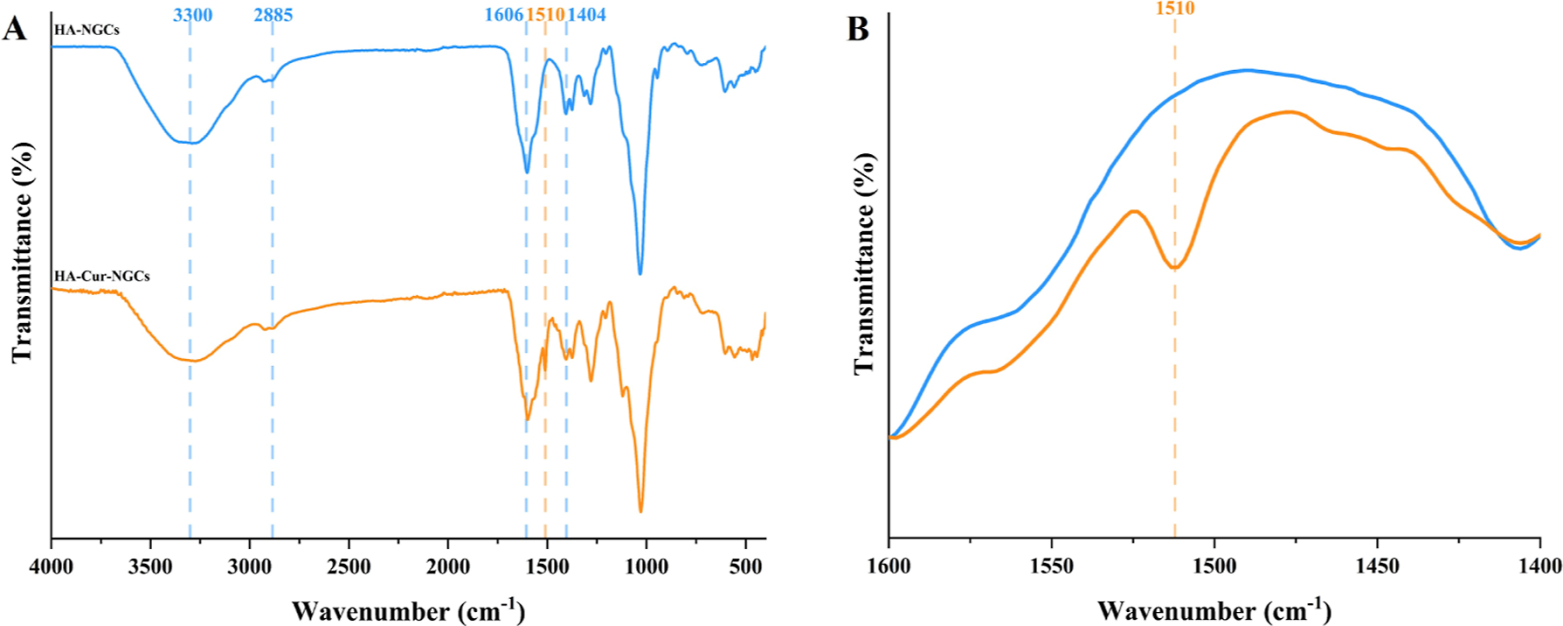
Chemical characterization of HA and HA-Cur nerve guidance conduits by Fourier transform infrared spectroscopy (FTIR). (A) FTIR curves of HA- and HA-Cur-NGCs. Blue dashed lines indicate HA characteristic peaks and orange dashed line indicate Cur characteristic peaks. (B) FTIR spectra of HA- and HA-Cur-NGCs at the wavenumber region between 1600 and 1400 cm^–1^ to better visualize characteristic peaks of Cur. Results shown are representative of *n* = 3 independent replicates.

In vitro drug release of curcumin from porous HA-NGCs (Figure 5) was evaluated under physiological conditions in PBS at 37 °C. A two-phase release profile was observed, resulting in a rapid release during the first 24 h and then slowing down to a relatively constant rate until 6 days of incubation, at which time the release rate reached a stable profile with a more sustained release. After the first hour of incubation, about 23% of the drug was released into the medium, and in the following hours it gradually increased to 50% in the first 24 h, due to the fact that the drug is retained in the matrix by adsorption, so the drug release is mainly controlled by the diffusion mechanism, releasing at a higher rate the curcumin particles adsorbed on the surface of the scaffold. After a few days, a slower and gradual release occurred, reaching a maximum cumulative drug release of about 60% after 6 days, displaying a sustained release profile that plateaued thereafter. This incomplete release (below 100% unless a cosolvent or surfactant is used) is expected and consistent with previous reports on curcumin delivery from hydrogel-based systems under PBS conditions.^34,35^ The limited aqueous solubility and strong hydrophobic character of curcumin constrain sink conditions and slow its diffusion through the matrix due to physical entrapment in the porous matrix of the highly cross-linked HA hydrogel. In addition, potential microcrystallization of curcumin within the conduit may further contribute to its partial retention. Based on Fick’s law the diffusion coefficient calculated from the slope of the release profile was 0.026.

**Figure 5:**
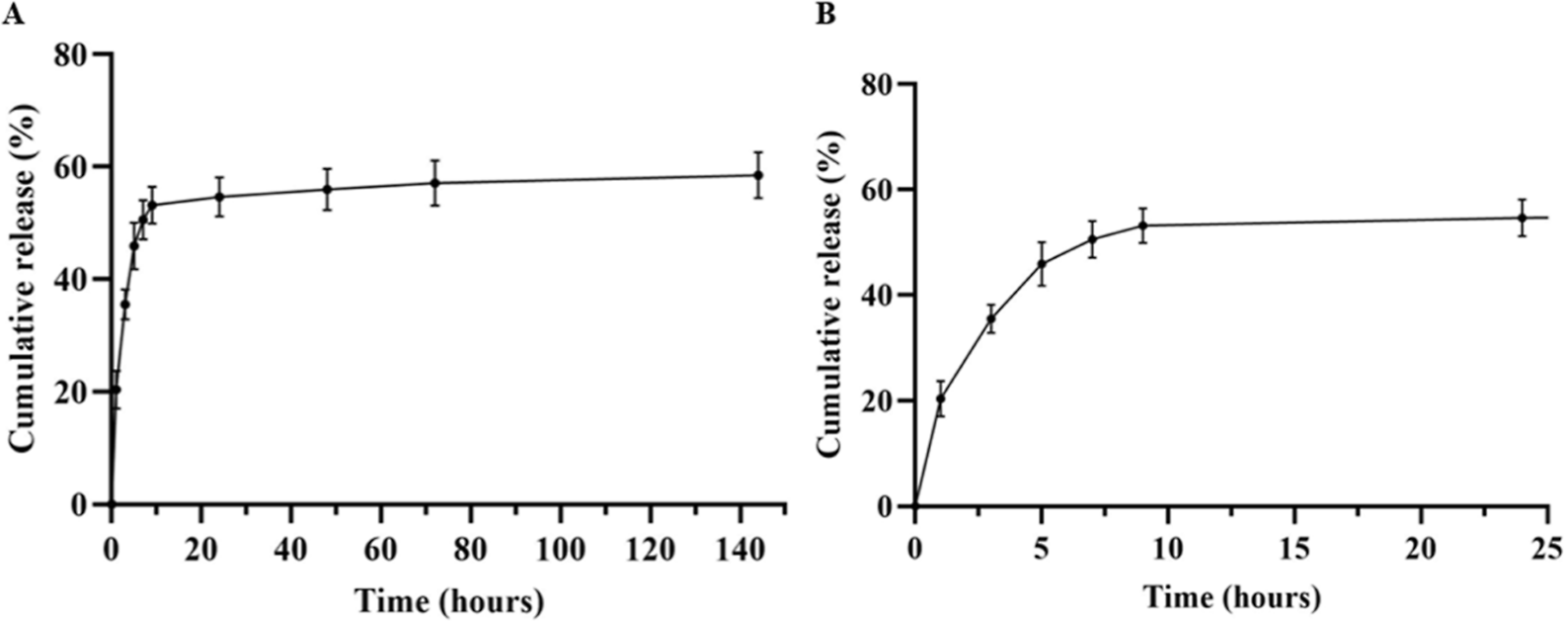
In vitro cumulative drug release study at 37 °C. (A) Curcumin release (%) profile from HA-NGCs at different time intervals in PBS determined by absorbance (*n* = 6). The detailed cumulative release over the first 24 h is shown in (B). Experiments were performed for up to 6 days. All measures were carried out in triplicate and all results are expressed as mean ± SD in terms of cumulative release (percentage with respect to the loaded drug) as a function of time. The diffusion coefficient is obtained by the slope of the release curve.

### 3.2 Cell Culture Assays

The biological effect of curcumin is dose-dependent and cell-type specific.^36,37^ Previous research has shown that curcumin at high concentration can trigger cell death in various cancer cells.^38,39^ On the other hand, low doses of curcumin induce proliferation in nervous system cells.^40,41^ Hence, it is crucial to determine the appropriate dosage of curcumin to be incorporated into the HA-NGCs and its effect on Schwann cells. To this end, hSCs were treated with different concentrations of curcumin (0–80 μM) for 24 h and their viability was determined by MTS assay. Schwann cells viability after curcumin treatment was dose-dependent (Figure 6). The IC_50_ value, representing the concentration of curcumin at which 50% of cell growth was inhibited, was determined at 16.47 μM. Curcumin concentrations between 0.5 and 7.5 μM increased hSCs proliferation, with 5 and 7.5 μM showing the highest cell viability. At higher concentrations, starting from 10 μM, a decrease in cell viability was observed. These findings, in combination with the curcumin release profile, guided the selection of the curcumin loading concentration for subsequent experiments, which was set at 10 μM. Although 7.5 μM curcumin produced the maximal hSCs cell viability in the preliminary assay (Figure 6G), a nominal concentration of 10 μM was used to load the conduits to compensate for the partial loss of curcumin expected during the preconditioning step in culture medium prior to cell seeding. This adjustment ensured that the effective concentration to which the cells were ultimately exposed remained close to the effective concentration.

**Figure 6:**
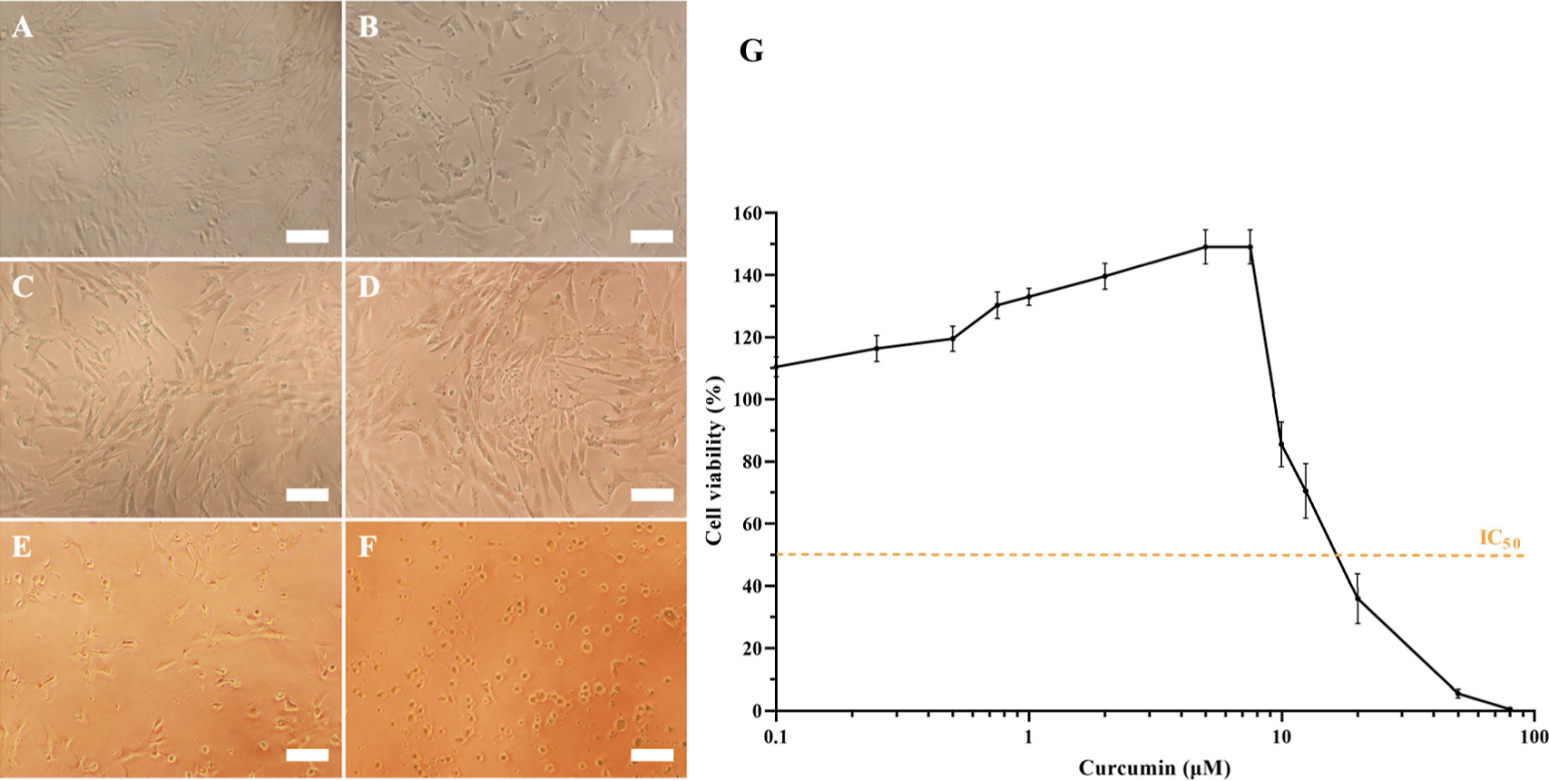
Curcumin concentration effect on Schwann cells: representative microscopic images of (A) control, (B) 0.1 μM, (C) 0.75 μM, (D) 2 μM, (E) 10 μM and (F) 20 μM. (G) IC_50_ curve of curcumin (0–80 μM) in Schwann cells by MTS assay. The viability of hSCs was measured after a 24 h treatment with the indicated concentrations of curcumin. Scale bar 100 μm. Data are expressed as mean ± SD (*n* = 4).

Schwann cells are integral to the neural repair process within the PNS, providing essential support for axon outgrowth as they secrete a plethora of neurotrophic factors^42^ and their regenerative effects both in vitro and in vivo have been documented.^13,43,44^ Therefore, the proliferation of SCs is essential for encouraging axonal regrowth and vital for nerve regeneration. To this end, the proliferation of hSCs cultured on HA-NGCs loaded with or without 10 μM of curcumin and filled with PLA microfibers, was assessed after 12 h, 1, 3, and 6 days of cell culture (Figure 7A). The results obtained from the MTS assays showed an increase of absorption with cell culture time for both HA-NGCs and HA-Cur-NGCs. However, absorption values on the curcumin-loaded conduits were higher, which means an increase in cell number and thus proliferation. Analysis of the MTS assay showed that at the early time points (12 and 24 h) there was no significant difference in metabolic activity between hSCs cultured on HA- and HA-Cur-NGCs (Figure 7A). These initial measurements, which primarily reflect early cell attachment, indicate that curcumin does not enhance initial Schwann cell adhesion to the HA matrix. In contrast, at days 3 and 6 the HA-Cur group exhibited a significant increase in metabolic activity, with an approximately 30% higher proliferative response compared to the control, demonstrating that curcumin’s effect emerges during the proliferation phase rather than during initial attachment.

**Figure 7:**
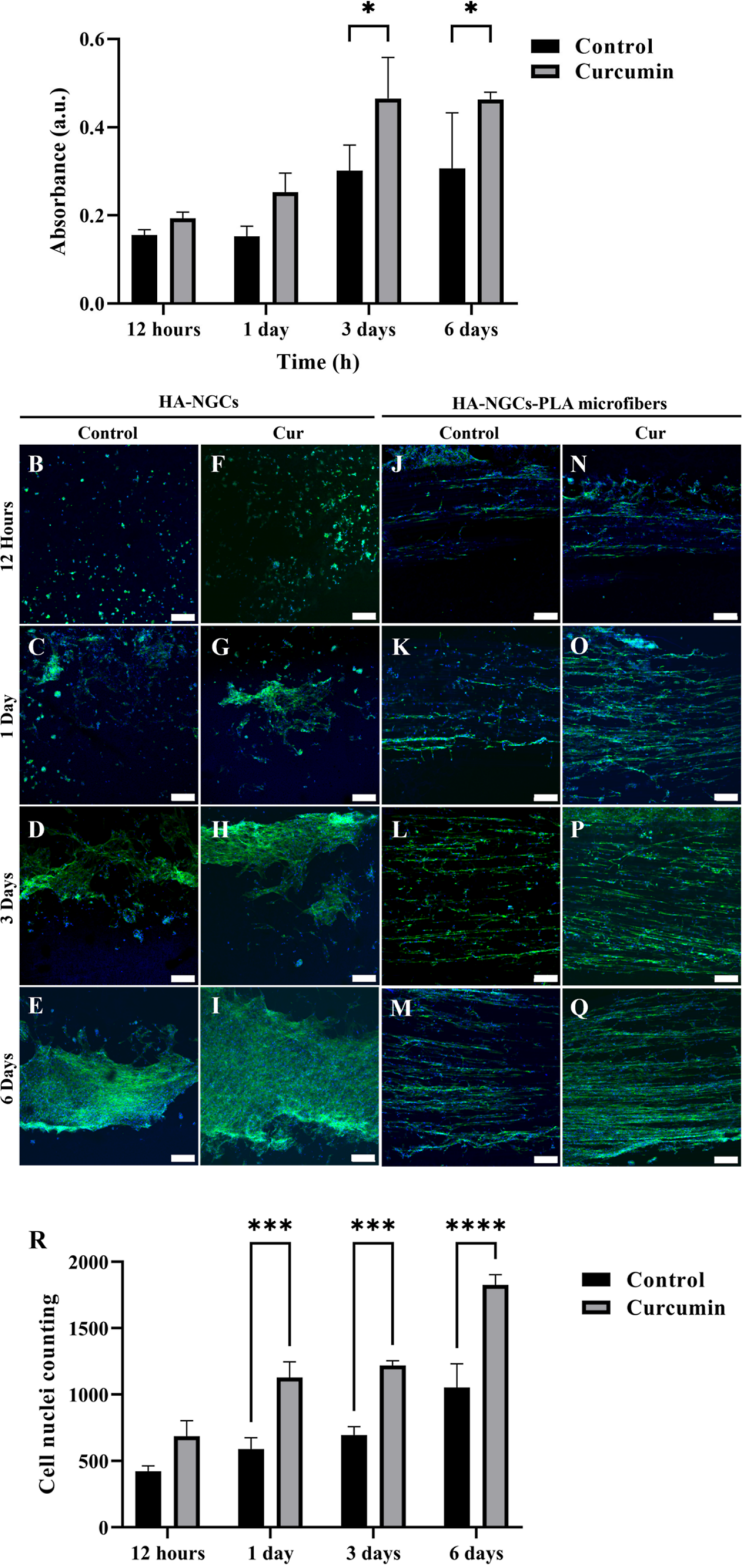
Human Schwann Cells proliferation on HA-NGCs and PLA microfibers. (A) MTS proliferation assays of hSCs on HA-NGCs at different culture times (12 h, 1, 3, and 7 days). Data are expressed as mean ± SD (*n* = 5). Statistical significance was determined by two-way ANOVA with Tukey’s multiple-comparison test; *p < 0.05. Representative confocal immunofluorescence images of hSCs seeded for 12 h, 1 Day, 3 Days and 7 Days on HA conduits (B–E) and curcumin-loaded HA conduits (F–I) and on PLA microfibers contained inside the lumen of HA-NGCs (J–M) and HA-Cur-NGCs (N–Q). The cytoskeleton is shown in green (phalloidin), and the nuclei are shown in blue (DAPI). Scale bar 200 μm. Quantification of Schwann cell nuclei on HA-NGCs containing PLA microfibers (R). Data represents mean ± SD (*n* = 3 images per condition). Nuclei were counted using the StarDist plugin in Fiji. Statistical analysis was performed by two-way ANOVA followed by Tukey’s multiple-comparison test; ***p < 0.001 and ****p < 0.0001.

These trends were reflected in the immunofluorescence images of hSCs cultured on HA- and HA-Cur-NGCs containing PLA microfibers. At 12 h, cells on HA conduits exhibited rounded morphologies and small aggregates (Figure 7B), consistent with the limited adhesion typically observed on hyaluronic acid.^45–47^ As culture time progressed, cells entrapped within the porous HA network secreted extracellular matrix (ECM) proteins, progressively improving local adhesion sites and enabling partial cell spreading (Figure 7C–E, days 1–6). In contrast, hSCs cultured on HA-Cur-NGCs displayed a higher degree of cell coverage and larger aggregates at comparable time points (Figure 7F–H), suggesting an enhanced proliferation rate. By day 6, a continuous layer of Schwann cells was observed over the HA surface (Figure 7I), consistent with the higher metabolic activity measured in the MTS assay, indicating that curcumin release contributed to improved cell proliferation.

Nuclei quantification (Figure 7R) further supported these findings: the number of nuclei was not statistically significant between groups at 12 h but increased markedly at later time points, confirming that the enhanced surface coverage observed in the fluorescence images is due to curcumin-mediated stimulation of Schwann cell proliferation, not improved initial adhesion. In summary, these results demonstrate that curcumin promotes sustained hSC growth, the increase in confluence and coverage resulted from enhanced Schwann cell proliferation supported by the curcumin-induced favorable microenvironment within the HA-NGCs.

In contrast, hSCs on PLA microfibers displayed a predominantly spindle-shaped morphology with a parallel orientation relative to the fiber’s axis (Figure 7J–Q) from the beginning, exhibiting a resemblance to bands of Büngner-like structures. At 12 h of cell culture, the orientation of the cells along the microfiber’s direction was achieved independently of the presence of Cur. However, as with the HA-NGCs’ lumen, the microfibers were fully covered with hSCs at an earlier stage in the HA-Cur-NGCs, confirming an increased proliferation rate. Mimicking in vitro structures that emulate Büngner bands has already been proven to enhance axonal extension.^48^ Therefore, the incorporation of PLA microfibers within the HA-NGCs to form pseudo Büngner bands will hinder axons from time-consuming undirected growth inside the conduit, thereby, facilitating and accelerating nerve regeneration.

Quantification of cell nuclei using the StarDist plugin revealed a trend consistent with the metabolic activity results obtained from the MTS assay. At 12 h, no significant difference in the number of nuclei was observed between hSCs cultured on HA- and HA-Cur-NGCs conduits, indicating comparable initial adhesion. However, from day 1 onward, the number of nuclei on HA-Cur samples increased significantly compared to the control group, with higher counts at 1 day, 3 days and 6 days, reflecting enhanced cell proliferation over time. This progressive divergence supports the notion that curcumin release from the HA-NGCs promotes a sustained proliferative response in Schwann cells, rather than merely a transient early effect.

## 4 Conclusions

To optimize the efficacy of nerve guidance conduits, it is essential to more closely replicate the intrinsic regenerative properties of native nerve tissue. Schwann cells play a pivotal role in axonal regeneration. Following injury, these cells proliferate, and form longitudinally aligned structures known as bands of Büngner, which serve as guiding pathways for regenerating axons. In addition to their structural role, Schwann cells secrete a wide array of growth-promoting factors.

In this study, the nerve guidance conduit we developed is composed of a hyaluronic acid conduit loaded with curcumin, with its lumen filled with PLA microfibers. We demonstrated that the sustained release of curcumin from the HA hydrogel significantly enhanced Schwann cell proliferation and PLA microfibers promoted their parallel alignment along their axis. This cellular organization within the conduit creates a supportive microenvironment conducive to nerve regeneration.

The HA-Cur-NGCs filled with PLA microfibers represent an enhanced NGC that may be employed in future studies to evaluate their capacity to accelerate axon regeneration across defects in an in vivo model of PNI.

## Data Availability

The data underlying the findings of this study are available within the published article.

## Abbreviations

PNIs: peripheral nerve injuries
NGCs: nerve guidance conduits
PLA: polylactic acid
SCs: Schwann cells
hSCs: human Schwann cells
Cur: curcumin
HA: hyaluronic acid
DVS: divinyl sulfone
PTFE: polytetrafluoroethylene
PCL: polycaprolactone
NaOH: sodium hydroxide
DMSO: dimethyl sulfoxide
PBS: phosphate-buffered saline
DMEM: Dulbecco’s modified eagle’s medium
FBS: fetal bovine serum
PFA: paraformaldehyde
NGS: normal goat serum
FESEM: field emission scanning electron microscopy
TGA: thermogravimetric analysis
FTIR: Fourier transform infrared spectroscopy
IC50: half maximal inhibitory concentration.
